# Meningococcemia complicated by myocarditis in a 16-year-old young man: a case report

**DOI:** 10.11604/pamj.2018.29.149.13107

**Published:** 2018-03-13

**Authors:** Rania Bouneb, Manel Mellouli, Haifa Regaieg, Senda Majdoub, Imed Chouchène, Mohamed Boussarsar

**Affiliations:** 1 Medical Intensive Care Unit, University Hospital F, Hached Sousse, Tunisia; 2 Faculty of Medicine of Sousse-Tunisia; 3 Department of Clinical Hematology, University Hospital Farhat Hached, Sousse; 4 Department of Radiology, University Hospital Farhat Hached, Sousse

**Keywords:** Neisseria meningitidis, myocarditis, recovery, shock

## Abstract

Fulminant meningococcemia is a relatively rare life-threatening disease caused by Neisseria meningitidis. The clinical presentation is varied, but, when associated with myocarditis, it carries a particularly poor prognosis. We report a case of a patient with fulminant meningococcemia who subsequently developed severe myocardial dysfunction and successfully recovered within a period of 7 days of hospitalization. A 15-year-old girl presented with headache, fever, body ache for 1 day and few ecchymotic rash over her body for 3 hours. Blood cultures confirmed infection with N. meningitidis. After 2 days in the hospital, the patient developed dyspnea, elevated jugular venous pressure and shock. The patient was managed with intravenous ceftriaxone, furosemide and norepinephrine. Over the next 4 days the patient rapidly improved. Meningococcemia complicated by myocarditis has an extremely poor prognosis with high mortality. Our case suggests that recovery from a severe myocardial dysfunction can occur rapidly within a few days. Prompt recognition and management in this case might have contributed to the patient's rapid recovery from myocarditis.

## Introduction

Fulminant meningococcemia is a relatively rare life-threatening disease caused by Neisseria meningitidis, and is perhaps the most rapidly lethal form of septic shock in humans. It differs from most other forms of septic shock by the prominence of hemorrhagic skin lesions. Meningitis, associated with fever, headache and nuchal rigidity, is the most common pathologic presentation of meningococcal disease and has a mortality rate of 5%-10%. Fulminant meningococcemia, as the name suggests, is the most severe form of infection by N. meningitidis [1,2]. Multiple factors have been identified that increase the transmissibility of N. meningitidis. Fulminant meningococcemia is associated with high mortality, with most deaths occurring in the first 24 hours [3]. The clinical presentation is varied, but when associated with myocarditis, carries a poor prognosis. We report a case of fulminant meningococcemia complicated by myocarditis.

## Patient and observation

**Figure 1:**
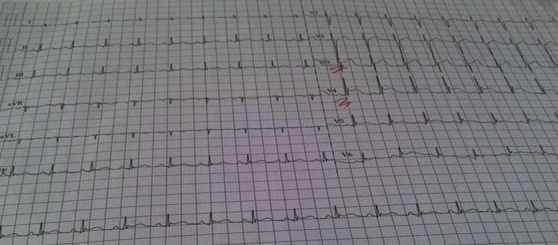
The electrocardiogram showed ST segment elevation in leads V1, V2, V3, DII, DIII and AVF

**Figure 2:**
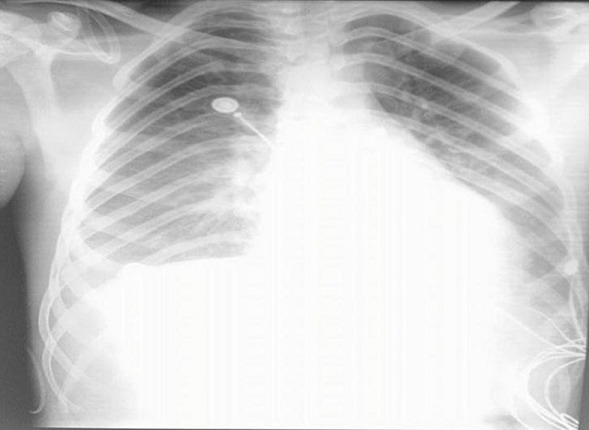
Chest X-ray showed pulmonary edema

**Figure 3:**
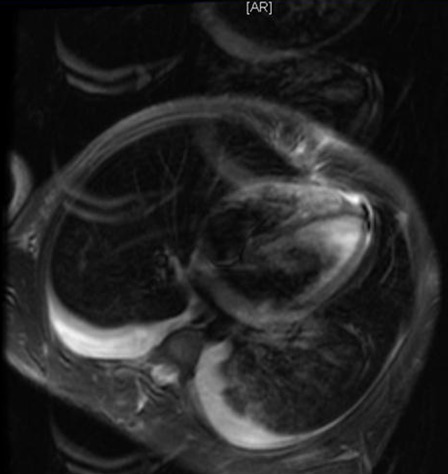
Cardiovascular magnetic resonance imaging performed at day 5 showing hypersignal T2 of the right ventricular

A 17-year-old man presented to the emergency department of Farhat Hached Sousse-Tunisia with complaints of headache, fever, body ache for 1 day. On presentation, she had a temperature of 39°C, heart rate of 120/minute, and blood pressure of 100/50 mm of Hg, cold extremities. Meningeal signs were present and numerous ecchymosis lesions over abdomen, arms and legs. Investigations revealed a platelet count of 70,000/µL and an international normalized ratio (INR) of 4. Blood cultures were positive for N. meningitidis. Lumbar puncture performed yielded a 2750 pleocytosis (100% polynuclear neutrophil) with elevated protein level (1,75g) and normal glucose in cerebrospinal fluid (CSF). CSF culture reveals a gram-negative diplococcus. The patient was treated with 4g intravenous (IV) ceftriaxone every 6 hours, vascular filling with saline 0.9%. Over the next day, she developed shortness of breath, worsening edema and shock. The electrocardiogram showed ST segment elevation in leads V1, V2, V3, DII, DIII and AVF (Figure 1). Troponin levels were slightly elevated at 75 ng/L; Total creatinine kinase (CK) was elevated 8547 U/L and an important cytolysis. Chest X-ray showed pulmonary edema (Figure 2) He is treated by norepinephrine. An echocardiography revealed global myocardial hyperkinesia with a lowered ejection fraction of 35%. The magnetic resonance imaging (MRI) confirmed the diagnosis (Figure 3). A diagnosis of myocarditis complicating acute meningococcemia was made. Over the next 3 days, the patient defervesced and gradually returned to her premorbid state. The patient was also given intermittent doses of furosemide (20 mg IV). She received ceftriaxone for a total of 4 days. Follow-up echocardiography revealed an ejection fraction of 60% with good mobility of the myocardium. She was discharged after a 7-day hospitalization.

## Discussion

Meningococcemia is a relatively rare disease caused by N. meningitides. The annual incidence in the United States and Europe has been suggested to be 0.35 cases per 100,000 and 1 case per 100,000, respectively [4]. The presence of a capsule is a major virulence factor and six capsular groups have been described as causing the majority of invasive disease (A, B, C, W-135, X, and Y). Among these, groups A, C, and W-135 are known to be common in Asia and Africa [5]. In epidemic settings in third-world countries, case fatality rates as high as 70% have been recorded [6]. Transmission of meningococci occurs through direct contact with respiratory droplets. The mucosa of the upper respiratory tract of human beings is the major reservoir and in most instances, the disease is acquired through close contact with asymptomatic carriers [5]. Clinical manifestations of N. meningitides infection are varied. Initial symptoms may mimic a viral disease; however, within a few days, more localized features develop in cases that develop meningitis, such as headache, nausea, stiff neck and vomiting [5]. The most characteristic clinical feature of fulminant meningococcemia is the development of petechial or ecchymotic rashes. However, meningococcemia can lead to complications such as massive adrenal hemorrhage, disseminated intravascular coagulation, arthritis, heart problems such as pericarditis and myocarditis, neurological problems such as deafness and peripheral neuropathy, and peripheral gangrene [1]. Our patient presented with fever, headache, with ecchymotic rash. The patient had evidence of meningitis. The course of illness in our patient was complicated by myocarditis. This complication is not recognized very frequently, but has been described before [7]. An autopsy study in children revealed the presence of myocarditis in 42% of 31 children who had died of meningococcal infection, though the extent of inflammation was mild in the majority [7] which suggests that myocarditis may be more common than is recognized clinically. Endomyocardial biopsy remains the gold standard for the diagnosis of myocarditis; lymphocytic infiltrates with myocardial necrosis are deemed to be characteristic [8].

We did not have the ability to perform right-heart catheterization or endomyocardial biopsy in our patient to make a definitive diagnosis of myocarditis. Also, there is a possibility that the elevated CPK and troponin were a result of myositis secondary to meningococcemia. However, chest radiograph, ECG signs, and echocardiographic findings of global hyperkinesia with a lowered ejection fraction suggest that the heart failure that developed was due to myocarditis rather than cytokine effect or fluid overload and the MRI confirmed diagnosis. In the present case, the diagnosis was made on the basis of the combination of clinical manifestations of myocardial dysfunction, increased serum cardiac troponin level, and typical echocardiographic and MRI abnormalities. MRI is a noninvasive method to diagnose myocarditis without the risk of endomyocardial biopsy. We chose in this case not to perform endomyocardial biopsy because we estimated that the benefit/risk balance was unfavorable and the MRI criteria for myocarditis were in accordance with the Lake Louise International Consensus [5]. The involvement of cytokines, and more particularly interleukin-6, has been suggested to explain the pathogenesis of myocardial dysfunction in meningococcemia. Current guidelines favor symptomatic management of myocarditis with the use of diuretics, ACE inhibitors and beta blockers along with treatment of the offending agent where applicable [8]. We chose to start the patient on captopril and furosemide for symptomatic management with careful monitoring of the blood pressure. There are no data to confirm if there is a benefit to using captopril in patients with myocarditis from meningococcemia. However, we opted to treat our patient cautiously with captopril as she had a clinical picture of worsening myocarditis with shock and could not afford treatment in the intensive care unit. Captopril is a potent vasodilator and helps left-ventricular failure by reducing the afterload on the heart muscle. Her recovery might have been beneficially influenced by the captopril. The unusual thing about our patient was her rapid and successful recovery within a span of a few days. Previous evidence has shown that resolution of clinical myocarditis is often prolonged and incomplete [9]. However, this report reaffirms the possibility of rapid resolution of severe meningococcal myocarditis, which has been seen before [10]. Pathologic evidence of myocarditis is more common than appreciated clinically in patients with meningococcemia. Prompt recognition and management in our patient may have contributed to her rapid recovery from myocarditis.

## Conclusion

Clinical myocarditis is an infrequently recognized complication of acute meningococcemia, and can lead to severe heart failure. Recovery from myocarditis may be delayed and take several months. Our case suggests that rapid resolution of clinical myocarditis is possible with early diagnosis and treatment.

## Competing interests

The authors declare no competing interest.
